# Feasibility, Acceptability, and Perspectives Regarding the Use of Activity Tracking Wearable Devices Among Home Health Aides: Mixed Methods Study

**DOI:** 10.2196/77510

**Published:** 2026-01-26

**Authors:** Ian René Solano-Kamaiko, Michael Dicpinigaitis, Melissa Tan, Irene Yang, Kexin Cheng, Ronica Peramsetty, Michelle Shum, Yanira Escamilla, Jennifer Bayly, Meghan Reading Turchioe, Ariel Avgar, Aditya Vashistha, Nicola Dell, Madeline R Sterling

**Affiliations:** 1 Cornell Tech New York, NY United States; 2 Weill Cornell Medicine New York, NY United States; 3 1199SEIU Funds New York, NY United States; 4 Columbia University New York, NY United States; 5 Cornell University Ithaca, NY United States

**Keywords:** home health aides and attendants, home health care, home care, worker well-being, frontline work, low-wage work, data governance, data advocacy, wearable activity trackers, passive sensing

## Abstract

**Background:**

Home health aides and attendants (HHAs) provide in-home care to the growing population of older adults who want to age in place. Despite their vital role in patient care, HHAs are an underserved and vulnerable population of health care professionals who often experience poor health themselves. Activity tracking devices offer a promising way to improve HHAs’ health-related awareness and promote health behavior change, particularly regarding physical activity and sleep quality, 2 areas in which the workforce struggles.

**Objective:**

This study aimed to understand how feasible it is for HHAs to use activity tracking devices and assess their perceptions of such devices for improving their health. Specifically, we conducted (1) a field study to assess the use, feasibility, and acceptability of these devices among HHAs and (2) a qualitative study to understand HHAs’ perspectives on and reactions to activity trackers on and off the job.

**Methods:**

We partnered with the 1199 Service Employees International Union Training and Employment Fund to conduct a field study with home care agency–employed HHAs working in New York City, New York. Participants wore activity tracking devices for 4 weeks that collected data on physical activity and sleep. The HHAs were subsequently interviewed on their experiences with and attitudes toward the devices and asked to reflect on personalized visualizations of their data to prompt them to think aloud. Quantitative data were analyzed using descriptive statistics. Qualitative data were analyzed using grounded theory.

**Results:**

A total of 17 HHAs participated; their mean age was 48.7 (SD 12.2) years, 15 (88%) were women, 11 (65%) identified as Black, 5 (29%) identified as Hispanic or Latinx, and they had worked as HHAs for a mean of 11.7 (SD 7.5) years. In total, 94% (n=16) of the HHAs wore their activity trackers for the full 28-day study period. Participants took a mean of 10,230 (SD 3586) daily steps during the study period and slept for a mean of 6.27 (SD 0.58) hours per night. Overall, 4 key themes emerged: (1) activity tracking devices enhanced participants’ health awareness by providing empirical data for self-reflection; (2) this increased awareness led to positive behavior changes, including setting and achieving health-related goals; (3) HHAs believed that these devices could improve not only their own health but also that of their patients through positive behavior changes; and (4) despite this optimism, participants emphasized that their ability to modify sleep and activity patterns was constrained by social and occupational determinants, with sleep improvements being particularly challenging.

**Conclusions:**

Our findings suggest that appropriately designed personal tracking interventions could offer a promising approach to supporting positive health-related changes in this historically overlooked workforce, potentially improving their well-being and the quality of care they provide to their patients.

## Introduction

Home health aides and attendants (HHAs) are an essential frontline workforce providing in-home care to a growing number of older adults who wish to age in place [1-3]. HHAs provide personal care to older adults, including assistance with activities of daily living and instrumental activities of daily living as well as medically oriented care (monitoring symptoms and vital signs and counseling patients on physical activity [PA], diet, and medication adherence), all while providing emotional support [4].

Despite their critical role in patient care, HHAs are an underserved and vulnerable group of health care professionals who often have poor health themselves [5-7]. Predominantly comprising middle-aged women from racial minority groups earning substandard wages (national median of US $12 per hour), this workforce numbers over 3.5 million in the United States and has a high burden of cardiovascular disease risk factors, including obesity, hypertension, hyperlipidemia, physical inactivity, smoking, and poor sleep [4,8,9]. Our recent national study revealed that HHAs’ health and health behaviors are notably worse than those of other frontline health care workers not employed in the home (ie, nursing homes and hospitals), with over one-third reporting fair or poor general health, nearly 40% being obese, and more than half being physically inactive [10]. Contributing factors to this include limited health insurance and opportunities to seek medical care, as well as little time to engage in positive health-promoting behaviors themselves. Left unaddressed, these occupational health disparities among HHAs could potentially harm them and compromise the quality of care they provide.

Activity tracking devices, also known as wearables, may be uniquely positioned to promote positive health behavior change in a workforce that has rarely been the focus of interventions and technology innovation. These devices, which include Fitbit trackers, Oura Rings, and others, show promise in potentially improving PA and sleep, 2 behaviors HHAs struggle to achieve optimal levels of, based on our prior studies [4,10]. Consistent with social cognitive theory and the theory of planned behavior, wearable devices allow users to visualize and reflect on their daily activity and sleep data, enhancing self-efficacy and promoting positive behavior change [11,12]. By improving their own health behaviors, HHAs may also model and encourage similar changes among their patients. To date, research on activity trackers in this context has primarily focused on their use among older adults [13,14], family caregivers [15,16], and facility-based care workers in international settings [17-19] and has never focused on HHAs, a growing workforce in constant contact with patients who also struggle to reach sufficient levels of PA and sleep. Furthermore, while these adjacent groups share caregiving roles, HHAs differ in that they are paid, agency-employed workers who face unique structural and occupational barriers to prioritizing their own health [20,21]. Therefore, studying HHAs extends existing work on caregiver- and worker-focused interventions by addressing a critical and understudied population within the US health care system.

To understand the potential for activity trackers to promote positive health behavior change, we conducted (1) a field study to assess the use, feasibility, and acceptability of these devices for daily use and (2) a qualitative study to understand HHAs’ perspectives on and reactions to activity trackers on and off the job.

## Methods

### Overview and Study Setting

We conducted this field and qualitative study from July 19, 2024, to October 11, 2024, in collaboration with 1199 Service Employees International Union Training and Employment Fund (1199SEIU-TEF), a labor management fund of the 1199 Service Employees International Union (SEIU) United Healthcare Workers East, the largest health care union in the United States [22]. The 1199SEIU-TEF provides education and training to more than 55,000 HHAs who are employed by over 50 licensed and certified home care agencies in New York City, New York [23].

### Study Participants and Recruitment

To be eligible, HHAs had to (1) be aged ≥18 years, (2) be English speaking, (3) own a smartphone (iOS or Android), and (4) be employed as an HHA in New York City. To recruit our intended sample of 25 to 30 study participants, 1199SEIU-TEF staff reached out to HHAs who had previously expressed interest in participating in research initiatives and invited them to take part in our study using standardized recruitment materials (email script and flyer). If potential participants were interested, staff referred them to the study team to be screened for eligibility. HHAs who were eligible for the study provided written consent. All aspects of this study took place in person at 1199SEIU-TEF headquarters in New York City.

### Data Collection and Study Procedures

Before conducting the field study, the research team met with participants to elicit their initial perspectives on activity tracking devices and the device form factor (wristband vs ring). From these conversations, we found that HHAs were enthusiastic about the potential benefits of activity trackers for increasing their health awareness. HHAs preferred wristband devices over rings as they felt that the wristband’s small screen would be easier to check discreetly during the workday than their phone and its form factor would not interfere with their daily tasks.

The field study involved giving HHAs Fitbit Charge 6 devices, which they were asked to wear for 28 days. Before providing the devices, we conducted a 1-hour onboarding session where participants were introduced to the research team; asked to respond to a few self-reported demographic questions (age, gender, race, Hispanic or Latinx ethnicity, educational level, and years of experience as an HHA); and guided through setting up the Fitbit device and mobile app, highlighting key features including activity and sleep tracking. After the participants created their Fitbit accounts, we walked them through the authorization process to allow the research team remote access to their data through the Fitbit application programming interface.

Participants were then asked to wear the Fitbit daily for 4 weeks, during which the research team contacted them weekly via their preferred method (SMS text message). Before the field study, we confirmed participants’ contact preferences (eg, SMS text message, email, or phone), and all preferred SMS text messages. Prior work has also supported this modality for communicating with HHAs [24].

SMS text message reminders prompted participants to wear the wristband, review their data, and contact the team with questions or issues. However, these reminders did not substantially affect engagement or acceptance. Most HHAs either ignored the messages or briefly acknowledged their receipt. In interviews, participants reported wearing the devices consistently because they valued the insights into their health and well-being, with SMS text messages serving primarily as a channel for troubleshooting or procedural clarifications (eg, how to access participant gift cards). Following the 28-day field study, we used the Fitbit application programming interface to collect participants’ data to create personalized visualizations showing each participant’s activities in relation to (1) the National Institutes of Health’s (NIH) recommended PA and sleep guidelines for adults and (2) their broader group of peers in the study.

Four investigators from the research team (IRS-K, MT, IY, and KC) then conducted 1-hour semistructured interviews with each participant to understand their experiences, data preferences, and insights into the wearable devices (Multimedia Appendix 1). Along with participants’ general perspectives, the investigators focused on device acceptability—as illustrated by participants’ attitudes toward the devices’ usability and perceived value. Following these discussions, we introduced the personalized visualizations of their data and prompted participants to engage in a think-aloud process [25]. Participants reflected from multiple perspectives: as individuals balancing complex personal and work lives, as members of a distributed peer group, and as part of a broader community advocating for improved working conditions. Interviews ended when thematic saturation was reached.

### Quantitative Data Analysis

Participant characteristics were analyzed using descriptive statistics. We collected participants’ Fitbit data, comprising 187 JSON files containing metrics on activity levels (sedentary, light, fairly active, and very active), step count, distance, heart rate, and sleep duration and stages (wake, light, rapid eye movement, and deep), each tagged with precise date and time stamps. We focused on sleep duration and step count and assessed use feasibility, defined as participants’ ability to consistently engage with the devices, by examining the date and time stamps associated with these metrics to determine whether the devices captured participants’ data on a daily basis.

We selected sleep duration and step count because these metrics were intuitive for participants (ie, required little explanation, were immediately interpretable, and were clearly actionable) and have clear policy relevance, such as the No More 24 campaign [26] advocating to end 24-hour HHA shifts. Furthermore, prior research indicates that Fitbit-derived sleep duration and step counts are reasonably reliable and accurate; systematic reviews and validation studies have found that Fitbit devices provide sleep duration estimates comparable to those of research-grade accelerometers [27,28], perform well in distinguishing wake from sleep [28,29], and meet acceptable validity and reliability standards for tracking PA in free-living conditions [30].

Participants’ Fitbit data (steps and sleep duration) were processed using exploratory data analysis methods in Jupyter Notebook [31]. As mentioned above, during the exploratory data analysis process, we generated data visualizations for the reflection interviews, which also incorporated auxiliary data such as participants’ sleep and work schedules along with the NIH’s PA and sleep recommendations to create benchmarks for optimal levels.

### Qualitative Data Analysis

Each participant interview lasted approximately 60 minutes, resulting in 17 hours of audio-recorded interview data. The interviews were professionally transcribed using NoScribe (noScribe.ai) [32], an open-source artificial intelligence tool run on the research team’s computers. We verified each transcript against the original recordings, correcting errors and redacting identifying information. Our analysis used grounded theory and, as such, used an inductive open coding approach [25] with 4 investigators (IRS-K, MT, IY, and KC). We established a baseline code set by jointly coding one interview and then validated our approach by coding a second interview in 2 separate pairs (IRS-K and KC as well as MT and IY), meeting to reconcile differences. The remaining 15 interviews were coded independently, with regular meetings to resolve disagreements and refine the coding scheme. Finally, we conducted affinity diagramming to synthesize these codes into high-level themes with representative quotations.

### Ethical Considerations

All participants gave informed consent to take part in the study, including consent for note taking, photo taking, audio recording of interviews, and collection of participants’ Fitbit data. Identifiable details in the data and features in participant images were removed to ensure participant privacy and confidentiality. Participants were compensated for their time with US $25 gift cards for each of the 3 components of data collection (preliminary discussions, field study, and final interview), for a total possible compensation of US $75 in addition to the Fitbit device, which they were allowed to keep. Cornell University’s institutional review board classified the study as expedited and reviewed and approved the project under protocol IRB0148598.

## Results

### Participant Characteristics

A total of 17 HHAs participated in this study. They had a mean age of 48.7 (SD 12.2) years, 88% (n=15) were women, 65% (n=11) identified as Black, 29% (n=5) identified as Hispanic or Latinx, and they had worked as HHAs for a mean of 11.6 (SD 7.5) years (Table 1).

**Table 1:** Sample characteristics (N=17).

Characteristic			Values
Age (years), mean (SD)			48.7 (12.2)
**Gender, n (%)**			
	Women	15 (88)	
	Men	2 (12)	
**Race, n (%)**			
	African American or Black	11 (65)	
	White	1 (6)	
**Ethnicity, n (%)**			
	Hispanic or Latinx	5 (29)	
**Educational level, n (%)**			
	High school diploma	3 (18)	
	Some college	3 (18)	
	Associate’s degree	4 (24)	
	Bachelor’s degree	5 (29)	
	Graduate degree	2 (12)	
Experience (years), mean (SD)			11.6 (7.5)

### Field Study Findings

Overall, 94% (16/17) of the HHAs wore their activity trackers for the full 28-day study period, with only 6% (1/17) wearing the device for 26 days, and 82% (14/17) indicated continued use after the study’s conclusion. As shown in Table 2, participants took a mean of 10,230 (SD 3586) daily steps during the study period, ranging from 3855 to 20,528 steps. In total, 53% (9/17) of the participants surpassed the NIH recommendations for 10,000 daily steps for healthy adults [33]. Table 2 shows that participants slept for a mean of 6.27 (SD 0.58) hours per night, ranging from 5.40 to 7.38 hours, with 18% (3/17) exceeding the NIH recommendations for 7 hours or more of daily sleep duration for healthy adults [34].

**Table 2:** Wearable device-measured mean steps and sleep duration among study participants (N=17). Comparison of mean daily step count and mean daily sleep (in hours) by participant. The National Institutes of Health recommends that healthy adults should walk 10,000 steps [33] and sleep 7 hours or more per day [34].

Participants	Daily step count, mean (SD)	Daily sleep (hours), mean (SD)
P1	3855 (2345)	6.59 (2.36)
P2	12,683 (4577)	6.58 (2.05)
P3	11,680 (2552)	5.82 (1.87)
P4	6867 (3705)	7.38 (1.58)
P5	7935 (5063)	5.75 (3.04)
P6	7133 (2782)	5.87 (1.95)
P7	20,528 (6384)	7.30 (0.99)
P8	10,262 (3442)	5.79 (1.56)
P9	10,739 (3386)	5.74 (1.66)
P10	11,928 (3351)	6.46 (1.30)
P11	11,133 (3217)	6.13 (1.95)
P12	9212 (3556)	6.36 (0.80)
P13	12,980 (7196)	5.40 (1.84)
P14	7026 (2396)	7.15 (1.85)
P15	7863 (1894)	5.64 (1.23)
P16	9063 (3976)	6.55 (1.17)
P17	13,024 (3123)	6.09 (1.76)

### Major Themes From Qualitative Interviews

A total of 4 major themes arose. Each theme is presented below with accompanying quotations that represent key concepts.

#### Theme 1: Activity Tracking Devices Were Feasible to Wear and Improved HHAs’ Health Awareness

Most HHAs found the use of the devices to be seamless and feasible to incorporate into their day-to-day activities. Many cited the personal value they derived from using the devices. Participant P9 shared their experience:

It’s becoming part of me, since one month I’ve been on it, so it’s like I’m getting accustomed to it.... I love wearing it because the information it provided...was very helpful to my life.

While many participants reported having a general awareness of their own health and well-being, a number stated how the activity tracker helped enhance their understanding of their own health. For instance, P4 said:

The difference is that with the Fitbit, I can see exactly how long I sleep or walk.... I know that sometimes I wasn’t sleeping, but I can [only] say maybe I slept five or six hours when I count. But with the Fitbit, it’s going to let you know exactly how long was the deeper sleep, the lighter sleep, and that stuff.

Participants also reflected on the fact that, after being shown their data, this informed their perceptions of their own health behaviors. For example, P5 reflected:

A lot of us are going to be surprised because in our mind, we’re sleeping a lot…. If this didn’t come about, I might have said, “oh, yes, I get enough sleep.” But this now is in fact showing me, “oh, girl, you’re not sleeping.”

This revelation was common among participants, who found that the tracking data offered a concrete way to reflect on their habits and gain a deeper understanding of how their work and home life impacted aspects of their well-being, such as sleep. P16 reflected:

When I look, sometimes I see I have four hours, 40 minutes of sleep.... I’m tired and everything is bothering me. But prior to the Fitbit, I didn’t realize what was bothering me.

#### Theme 2: Activity Tracking Devices Helped HHAs Set and Achieve Health Goals

Many participants shared how increased awareness of their health led to positive changes in behavior. Several reported adopting healthier habits because of heightened awareness of their sleep patterns. Examples they shared included limiting television and phone use (ie, screen time), adjusting their bedtime and wake-up times, and incorporating relaxation techniques such as warm showers and listening to calming music. For example, P6 explained:

When I watched my sleep time (on my device), I was sleeping between three and five [hours].... [Now I] try and see if I could fall to sleep earlier.... I started getting eight hours, seven hours. I started trying to focus on going to sleep now, [no] TV, [no] phone.

Reflecting on their activity data also had a motivating effect for many participants, helping them re-evaluate their existing behaviors and adopt new habits. Several mentioned that these changes had a compounding effect, where improvements in one area, such as increased walking, made them feel better and motivated them to walk even more. P5 shared their experience:

If I’m out there...for five, six minutes and I don’t see the bus, I’m going down the road. [Before] I wouldn’t do that. I’d stay there and wait for the bus. But it [the Fitbit] really encourages me now to walk. And as I said, the more I walk, the lighter I feel. The more encouraged I feel.

Other participants highlighted how greater health awareness helped them set and achieve specific health goals. For example, P3 discussed how she aimed to improve her PA at work to meet the step count goal (which was preprogrammed into the device). She said:

Sometimes I go to work, when I see I didn’t have the number of steps required, when I put my patient to bed, I went out to finish the steps. To have the score. So that was very interesting because I never did it before.

#### Theme 3: Activity Tracking Devices May Have Potential to Improve the Health of HHAs and Their Patients

HHAs were acutely aware of the negative impacts of poor sleep on their health and well-being. Beyond personal health improvements, they expressed concerns about their ability to perform their high-stakes job, which demands constant vigilance, when sleep deprived. For instance, P5 highlighted this challenge, stating:

If you don’t get enough rest, you cannot function effectively.... So, you as a [home care agency] admin, you want your workers to perform effectively. What will you do now for us so we can get adequate sleep?

At the same time, several HHAs were optimistic about the potential of activity tracking devices to enhance both their personal health and the health of their patients while on the job. For example, P8 said:

If you get a case that will let you [and] the patient walk. So the two of you can walk around and do more exercise.

#### Theme 4: HHAs’ Abilities to Make Sleep and Activity Changes Were Impacted by Social and Occupational Determinants

HHAs reported that certain factors related to their social environments or working conditions impacted their health and made it challenging to make desired health changes. For example, participants working live-in cases or overnight shifts mentioned that their sleep schedules were dictated by patients’ demands. P17 explained:

In live-in case we, by the law, [we] are supposed to sleep five hours not interrupted, but it’s not possible.... You can’t sleep this amount of hours.... They [patients] put TV on high volume and you can’t sleep.

This demonstrates the limited control that HHAs have over their sleep schedules. Additionally, HHAs expressed the need to remain vigilant, attending to patients’ needs and staying alert for emergencies. This constant hypervigilance negatively affected their ability to relax and sleep during overnight shifts.

HHAs also noted the challenges of nontraditional work schedules. Shift work, subject to frequent changes based on patient needs, made it difficult for many to regulate their sleep, even on days off. For instance, P4 described the stability of working with the same patient vs their current unpredictable schedule:

Like last year or years before, I have a stable patient. I know that I was working four days a week with the same patient.... I know exactly what was my schedule at that time. But right now, I have no fixed schedule.

Others, such as P15, faced difficulties regardless of their work schedule (daytime or nighttime), expressing difficulties balancing responsibilities in addition to work, such as school, family, and more, which impacted their ability to stay healthy:

Once I’m at home, I want to like catch up [on] my [school] assignments or cause currently I’m doing my internship as well. I still have to keep up with my personal life, my kids, my husband.... I have three kids and they are just like babies.... So when I’m at home, I think I tend to do more cause, if I can sleep, they have to like sleep before I can get to do anything.

Beyond sleep, HHAs also felt that their PA was often dictated by patients’ needs. P14 explained:

My patients, some of them, they want company.... They say, “let’s watch a movie, or let’s go to the theater,” and they call an Uber. So you don’t walk that much. You walk during the time that you’re going to work, and then you go home.

While some HHAs found that their PA was limited by their cases, others felt that their activity levels were more malleable. Many developed strategies to increase exercise, such as walking instead of taking the bus, neighborhood walks on days off, and encouraging patients to go outside for exercise with them during downtime.

## Discussion

### Principal Findings

To our knowledge, this is the first study to examine how activity tracking devices influence HHAs’ awareness of their health and well-being using empirical data from the devices and qualitative reflection interviews with participants. Our findings demonstrate that the use of activity trackers was both feasible and acceptable among HHAs. Feasibility was reflected quantitatively by participants’ ability to consistently engage with the devices, with 94% (16/17) wearing them for the full 4-week field study period. Acceptability was shown qualitatively through participants’ reflections during follow-up interviews, where they expressed positive attitudes toward the devices’ usability and perceived value for increasing health awareness. This level of engagement is noteworthy as previous research has shown lower participant adherence to consumer wearables [35]. The high engagement observed among HHAs in this study suggests that activity tracking interventions may be particularly effective for supporting the health and well-being of HHAs more broadly, although further research is necessary to validate this assumption.

Additionally, the Fitbit Charge 6’s wristband form factor and small screen provided participants with a discreet, functional user experience. Beyond use and feasibility, HHAs not only found that the devices offered them more awareness of key aspects of health—PA and sleep—but even in a short time, they reflected that they were empowered to make positive behavior changes because of wearing the devices. Finally, we found that the devices may have health benefits not only for HHAs but also for the patients for whom they care.

Our study builds on scientific literature in a few key ways. First, while many studies have focused on the acceptance and impact of wearables among older adults [13,14], few have addressed their use among family caregivers and facility-based health care workers [15-19], and even fewer have examined these applications with HHAs [36].

For example, several studies conducted in Finland and Norway have examined the use of wearables among family caregivers and facility-based home care nurses, nursing assistants, and occupational therapists [17-19]. However, these studies often relied on technologies such as accelerometers [17,18] and electrode-based monitors [19], which can be difficult to set up and may not offer real-time or easily interpretable health feedback for lay users, unlike consumer-friendly devices such as Fitbit trackers.

To date, there has been one prior case study in which Fitbits were deployed among caregivers at the SEIU 775 Benefits Group in Washington state [36]. The study found that using Fitbit devices, combined with health coaching, resulted in improvements in health behaviors over a 4-month period. These improvements included increased PA (measured via step counts and active minutes), weight loss, and reduced blood pressure. The findings were based on multiple blood pressure readings, self-reported weight loss, and recorded activity data from the devices. However, this was a pilot program conducted by SEIU 775 Benefits Group in collaboration with Fitbit, Inc (a Google subsidiary), resulting in a self-authored “case study” rather than a peer-reviewed journal article. While the case study presents promising findings, its details and depth are limited due to its intended audience. Our study builds on this preliminary work by outlining a detailed and robust study methodology focused on Fitbit devices for improving HHAs’ awareness of their health and well-being. Additionally, our study incorporates both wearable data and qualitative reflections, aimed at providing a deeper understanding of how HHAs navigate barriers to improving their health and how tracking devices may help support these efforts.

Prior research has shown that wearable devices support health monitoring and facilitate behavior change, particularly in PA and sleep [13,16], and our study extends the literature to HHAs. In doing so, it highlights how these devices have the potential to improve the health of a vulnerable population and shape workplace dynamics and patient care within home care settings. Importantly, we found that wearables can not only track behavior but also offer a simple and feasible way to readily influence workplace routines, patient interactions, and broader occupational health strategies in home care settings. This finding is notable as previous research has shown that influencing behavior change is often challenging and may reflect HHAs’ perception of being in control of their own health [37].

In recent years, there has been a growing call for better understanding and improving the health of HHAs [4,7,9,10]. However, empirical studies that aim to improve their health behaviors and health are scarce. Our study is a first step toward this through harnessing technology and offering HHAs real-time access to their own health data. While we found striking benefits, it is also important to note the challenges. For example, while these devices can offer HHAs personalized feedback and enhance user motivation and self-efficacy [13,16], we also found that external and structural barriers in the environments in which the HHAs live and work can limit the extent to which HHAs can act on their health feedback, making it difficult to prioritize meaningful and sustainable behavioral health changes. For example, unpredictable work schedules, multiple jobs, and familial obligations all left them with limited time and resources to obtain sufficient sleep.

Nevertheless, HHAs found the intervention valuable, using wearable devices to self-monitor, reflect on current health habits, and make changes to their behaviors. Some participants adjusted their sleep routines, such as modifying bedtimes and reducing screen time before sleep, whereas others increased their PA in response to real-time device feedback. Consistent with social cognitive theory and the theory of planned behavior, these self-directed changes suggest that wearables can increase health awareness and motivation to change, representing a promising approach to promote health behavior change among HHAs [11,12].

Although structural barriers such as power asymmetries among workers, patients, and agencies; low wages; and isolated work environments limit HHAs’ ability to improve their health and well-being, wearable data could be integrated into existing peer support programs that aim to reduce worker isolation and strengthen community ties [38,39]. For example, in our prior studies, we have found that HHAs are more likely to engage in PA if they know peers or their clients are engaged as well. Our findings in this study may help bolster and extend these efforts, which have largely relied on HHAs’ self-reported experiences. Aggregated wearable data could more concretely illustrate the challenges that HHAs face, enrich qualitative accounts, and guide the design of peer support programs by grounding discussions and activities in workers’ collective experiences to identify strategies for navigating or mitigating these structural constraints.

Notably, the findings also suggest that PA may be more modifiable than sleep for the HHAs and have clearer positive implications for their patients. That is, several participants found that wearing their activity trackers reminded them of the importance of PA on the job and motivated them to spend more time walking with their patients to increase both their step counts. This is an important observation as previous research has identified challenges in improving PA among older adults receiving home care [40] and researchers have suggested that activities such as walking are valuable for promoting PA outside of structured exercise programs [41].

In future studies, this relationship warrants investigation. Key questions include the following: does increasing HHAs’ step count in turn increase their patients’ PA and mobility and reduce the risk of falls? Does increasing time spent together moving foster closer relationships between HHAs and their patients through shared activity? Our work has previously found that higher levels of mutuality between HHAs and patients result in higher job satisfaction among HHAs [42], and this too could be formally examined after introducing technology. Another important area for exploration is the affordability of wearable devices for a workforce with limited disposable income (eg, the Fitbit Charge 6 costs approximately US $150 as of this writing). If these devices are shown to significantly impact worker and patient outcomes, they may be justified for inclusion in future workforce wellness programs. Alternatively, existing devices such as smartphones could offer a more cost-effective means of collecting data on PA and sleep. However, unlike wearable devices, smartphones are not continuously worn on the body, which may limit the accuracy of the data they capture. Regardless, the feasibility of using smartphones should be further examined.

Beyond improving personal health, these devices may help further center workers’ experiences by introducing an additional layer of granular quantitative data to complement workers’ qualitative narratives. This collection of data sources may help bolster ongoing advocacy efforts, such as supporting improving wages or better working conditions. For example, collective data can strengthen labor union and worker advocacy campaigns such as the push for Fair Pay for Home Care [43], which advocates for livable wages and job stability for HHAs. Similarly, the No More 24 campaign [26], which seeks to end the harmful practice of requiring HHAs to work 24-hour shifts, where HHAs work for 24 hours but are only paid for 13 hours under the premise that they receive at least 5 hours of uninterrupted sleep and other breaks, is directly relevant to HHAs’ poor sleep levels. Our research suggests that tracking HHAs’ activities could generate data-driven evidence on sleep patterns and quality, which, when combined with worker-centered reflections that contextualize these data, could provide compelling arguments for policymakers and bolster organizational advocacy efforts. Future research should explore how to responsibly manage and share HHAs’ collective data in ways that center their needs and experiences to improve their health and well-being.

### Limitations

We acknowledge that our study has several limitations. We conducted a small-scale, short-term empirical mixed methods study in a single geographic location, New York City. Further research is needed to understand how our findings might generalize to larger samples and other locales, including rural and non-US settings, particularly those where driving is a more frequent mode of transportation than walking or public transit. Participants were recruited through their affiliation with a labor management sector of a health care union and may have been more motivated to engage with the wearable devices than nonunionized HHAs, which could confound our findings and limit generalizability to the broader HHA workforce.

Our study did not ask separate questions regarding sex and gender or race and ethnicity, which may obscure participants’ intersectional experiences. Future work should aim to include these perspectives. Additionally, researchers should investigate the results of a longitudinal study to understand how participants’ experiences might change over time and how they objectively impact the patients they serve. Our study also has limitations inherent to qualitative interviews, including potential participant response bias [44]. Further research might explore techniques such as journaling and remotely distributed surveys to decrease the impact of researchers’ positionality.

### Conclusions

Our findings suggest that personal tracking devices offer a feasible and acceptable approach to supporting positive health-related changes in this historically overlooked workforce. These devices have the potential to improve both the health behaviors and well-being of HHAs and, potentially, of the patients for whom they care. These concepts should be formally tested in future clinical trials.

## Appendix

Multimedia Appendix 1 Qualitative interview guide.

## Abbreviations

1199SEIU-TEF: 1199 Service Employees International Union Training and Employment Fund
HHA: home health aide or attendant
NIH: National Institutes of Health
PA: physical activity
SEIU: Service Employees International Union

## Data Availability

The datasets generated or analyzed during this study are available from the corresponding author on reasonable request.
