# Supplementary material for: Feasibility, Acceptability, and Perspectives Regarding the Use of Activity Tracking Wearable Devices Among Home Health Aides: Mixed Methods Study
Source: J Med Internet Res. 2026 Jan 26;28:e77510. doi: 10.2196/77510 (PMC12887562; doi:10.2196/77510)
Supplement: Multimedia Appendix 1 [file jmir_v28i1e77510_app1.docx]

## Qualitative Interview Guide

### Introduction

Hi ______, thanks for participating in our study! Today we are going to discuss your experiences wearing the Fitbit and the information that the Fitbit collected on you. Do you have any questions before we begin?

Great! Before we get started, we know you previously signed a consent form but we want to double check that you consent to participating in our study?

Is it OK if we record our conversation for research purposes?

### Experience Questions

- What did you enjoy most about wearing the Fitbit? The least?
- What was the most useful information to you? The least?
- Did you learn anything about your own habits from wearing the Fitbit?
  - What did you not know, that now you do know about yourself — If anything?
- How often did you find yourself looking at the Fitbit or phone app?
  - What were you mostly checking? Why?
- Did you experience any challenges using the Fitbit? Was anything confusing?
- Did the Fitbit information cause you to change anything about your day?
  - E.g., did you do things in order to take more steps? Or get more sleep?
- Are you currently still using the Fitbit? Why or why not?

### Data Questions

#### Individual Sleep

- What do you notice about the data? Does anything stand out to you?
  - Do you notice any differences between your sleep on the days you work versus the days you have off?
- Is there any sleep information that is surprising to you or unexpected?
- Do you think you sleep as much as other HCWs?
- Do you think the information captured was ever wrong? Why or why not?
  - Is there anything missing in this information?
- Did you try to change your behavior based on the information?
  - E.g., your bedtime? Trying to go to bed earlier?
  - If so, did it work? What was the result?
  - If not, do you think having this information changed anything? If, so what?
- Would this sleep information be helpful to you? Why or why not?
- After viewing this information would you want to change anything about your sleep?
  - Why or why not?
  - How would you make these changes?

#### Peer Group Sleep

- What are your thoughts about seeing other HCWs average sleep information?
  - What do you think this information says about HCWs sleep?
  - Is this information helpful to you? Why or why not?
  - Do you think this information could be used by HCWs? Agencies? 1199SEIU?
- Does seeing other workers’ information change your opinions about your own sleep?

#### Individual Steps

- What do you notice about the data? Does anything stand out to you?
  - Do you notice any differences between your activity on the days you work versus the days you have off?
- Is there any activity information that is surprising to you or unexpected?
- Do you think you walk as much as other HCWs?
- Do you think the information captured was ever wrong? Why or why not?
  - Is there anything missing in this information?
- Did you try to change your behavior based on the information?
  - E.g., the amount you exercised? Trying to walk more by getting off the subway one stop before?
  - If so, did it work? What was the result?
  - If not, do you think having this information changed anything? If, so what?
- Would this activity information be helpful to you? Why or why not?
- After viewing this information would you want to change anything about your activity?
  - Why or why not?
  - How would you make these changes?

#### Peer Group Steps

- What are your thoughts about seeing other HCWs average steps?
  - What do you think this information says about HCWs activity?
  - Is this information helpful to you? Why or why not?
  - Do you think this information could be used by HCWs? Agencies? 1199SEIU?
- Does seeing other workers’ information change your opinions about your own activity?

### General Questions

- Would you be interested in using a Fitbit for work or your personal life? Both?
- Is there any information that you did not see, that you would have liked to see? Why?
- How comfortable are you with sharing your sleep and activity data with your agency?
  - Other home health aides?
  - 1199SEIU?
  - Policymakers?
- In what ways could this information be useful for your agency?
  - Other home health aides?
  - 1199SEIU?
  - Policymakers?
- Is there anything you absolutely do or do not want collected by the Fitbit?
- Do you have any concerns about sharing this information?

### Conclusion

Amazing! Thank you so much for your time and thoughtful responses. This is the end of our study but we are happy to answer any questions you may have before we end our session today.
